# A review of epidermal growth factor receptor ligands in glucose homeostasis

**DOI:** 10.3389/fendo.2026.1826175

**Published:** 2026-05-29

**Authors:** Ka-Ying Chan, Chu-Jun Deng, Xi Chen, Yin Cai, Shiqi Jia, Chi-Ming Wong

**Affiliations:** 1 Department of Health Technology and Informatics, The Hong Kong Polytechnic University, Hong Kong, Hong Kong SAR, China; 2 Institutes of Biomedical Sciences, Inner Mongolia University, Hohhot, Inner Mongolia, China

**Keywords:** amphiregulin (AREG), betacellulin (BTC), epidermal growth factor, epidermal growth factor receptor (EGFR), epigen (EPGN), epiregulin (EREG), heparin-binding EGF-like growth factor (HBEGF), transforming growth factor-alpha (TGF-a)

## Abstract

The epidermal growth factor receptor (EGFR/ErbB1) is a critical regulator of cellular proliferation and differentiation. While its role in oncology is well-characterized, its contribution to glucose homeostasis has emerged as a significant area of metabolic research. This mini-review synthesizes current evidence regarding the seven EGFR ligands—EGF, TGFα, amphiregulin, HB-EGF, betacellulin, epiregulin, and epigen—in regulating beta cell mass, insulin secretion, hepatic gluconeogenesis, and peripheral insulin sensitivity. We critically evaluate the transition from mechanistic *in vitro* studies to preclinical rodent models, while acknowledging the current lack of human clinical validation. Furthermore, we discuss the metabolic paradox of EGFR tyrosine kinase inhibitors (TKIs) and the safety considerations regarding the mitogenic potential of these ligands. By highlighting emerging evidence and structural innovations such as chimeric ligands, this review provides a roadmap for future translational research in the EGFR/ErbB metabolic signaling axis.

## Epidermal growth factor receptor and its ligands

Epidermal growth factor receptor (EGFR) also known as erythroblastic leukemia viral oncogene homolog B1 (ErbB-1) or human epidermal growth factor receptor 1 (HER1), belongs to the ErbB family of transmembrane receptor tyrosine kinases (RTKs) (1). Since its landmark discovery by American biochemist Stanley Cohen in 1962 (2), EGFR has been identified as a key mediator of cellular processes in both healthy tissues, such as epithelial cells and fibroblasts, and in oncology. For this seminal discovery, Cohen was awarded the 1986 Nobel Prize in Physiology or Medicine.

Currently, besides Epidermal Growth Factor (EGF), six ligands can bind to and activate the EGFR, namely transforming growth factor-alpha, amphiregulin, heparin-binding EGF-like growth factor, betacellulin, epiregulin and epigen (3). Upon ligand binding to the N-terminal extracellular domain, EGFR undergoes a conformational change that activates its intracellular tyrosine kinase domain. This triggers a cascade of downstream signaling pathways, including MAPK/ERK and PI3K/AKT, which control cell survival, proliferation, and differentiation (4). Dysregulation of these pathways is a hallmark of various malignancies, where aberrant EGFR activation promotes tumor growth and metastasis (5). Consequently, EGFR has become a primary target for therapeutic interventions, including monoclonal antibodies and tyrosine kinase inhibitors (TKIs) such as rociletinib and osimertinib (6, 7).

## Epidermal growth factor receptor in the regulation of glucose homeostasis

However, it is worth noting that some of the EGFR TKIs, such as rociletinib, have been associated with dose-limiting hyperglycemia in humans. Rociletinib specifically targets mutated forms of EGFR, including exon 19 deletions, L858R, and T790M mutations, but not exon 20 insertions (8). Patients receiving rociletinib treatment have reported the need for dose reduction, administration of oral antihyperglycemic agents, or both, to manage hyperglycemia while continuing therapy. This suggests that chronic treatment for hyperglycemia related to EGFR TKIs targeting T790M may be necessary (6).

However, in rodent models and clinical reports of type 2 diabetes (T2D) have demonstrated that the first generation of EGFR inhibitors gefitinib and erlotinib can reverse hyperglycemia, improve in glucose tolerance and insulin sensitivity (9). These observations highlight the potential involvement of EGFR in the pathophysiological aspects of glucose homeostasis.

As recent evidence suggests that EGFR signaling is also involved in the fine-tuning of energy metabolism. To provide a systematic overview of this role, we searched PubMed and Web of Science for studies published between January 1990 and August 2024. Search terms included “EGFR ligands,” “ErbB1,” “glucose homeostasis,” and “insulin sensitivity,” cross-referenced with specific ligands (e.g., “EGF AND diabetes”). We prioritized peer-reviewed original research providing mechanistic insights and excluded studies focusing exclusively on cancer cell metabolism lacking systemic physiological context.

## The impact of EGF and EGFR on insulin secretion

Intravenous glucose administration elevates systemic EGF levels, which triggers rapid, dose-dependent insulin secretion from MIN6 cells and mouse islets (10). Its effect occurs within one minute and is completely abrogated by the EGFR inhibitor AG1478, confirming the receptor’s essential role (10). Mechanistically, acute EGF administration stimulates beta cells via calcium influx and phospholipase D (PLD) activation (10). Furthermore, membrane protease prostasin (PRSS8) has been proposed to regulate this process by modulating the shedding of endogenous EGF in response to glucose (11). These findings suggest a functional link between EGF-induced and glucose-stimulated insulin secretion (GSIS), implying that systemic EGF levels may be physiologically regulated by nutritional status or feeding conditions. The effect of short-term fasting/refeeding on epidermal growth factor content in the gastrointestinal tract of suckling rats was reported (12). It remains to be determined whether these localized gastrointestinal changes translate to systemic fluctuations in humans and how they impact long-term metabolic homeostasis.

## The impact of EGF on beta cell mass

Beyond acute insulin secretion, EGFR activation promotes beta cell proliferation and survival. Alloxan-treated mice are a widely recognized model for insulin-dependent diabetes mellitus due to the selective destruction of pancreatic beta cells (13). Alloxan induces beta cell death by generating reactive oxygen species, resulting in a significant loss of insulin production and persistent hyperglycemia (14). In alloxan-induced diabetic mice, a combination of gastrin and EGF restored normoglycemia and increased islet mass within three days (15). Mechanistically, EGF enhances the stability of the anti-apoptotic protein surviving and activates the transcription factor ETS2, which suppresses miR-124a to sustain insulin production (16, 17). However, these findings are primarily limited to *in vitro* (MIN6 cells) and *ex vivo* islet studies; human data correlating endogenous EGF levels with diabetes progression remain observational and inconclusive. Notably, EGFR signaling also mediates pregnancy-induced beta cell expansion in mice (18). While these rodent findings provide a robust mechanistic framework, human data remain largely observational, and it is unclear if systemic EGF fluctuations are a primary driver of glucose homeostasis in humans.

To investigate the impact of EGF on glucose homeostasis in neonates, an *in vivo* study focused on the early neonatal period of rats (19). The study revealed that EGF treatment resulted in increased levels of hepatic glycogen, elevated concentrations of plasma insulin, and a higher proportion of beta cells within the islets of Langerhans. Additionally, the concentration of plasma glucagon was reduced in the group treated with EGF (19). These findings suggest that EGF influences glucose metabolism during the early neonatal period by modulating the pancreatic endocrine system, leading to a shift from glucose catabolism to glucose anabolism. Notably, this was reflected in an elevated insulin-to-glucagon molar ratio, indicating the significant impact of EGF on glucose metabolism (19).

## The impact of EGF on hepatic gluconeogenesis

The role of EGF in hepatic glucose production appears context-dependent. Early studies reported that EGF stimulated gluconeogenesis in isolated rat hepatocytes by inactivating pyruvate kinase (20–22). However, a subsequent study found that EGF decreased both the basal- and the glucagon-stimulated gluconeogenesis from lactate alone or from a high lactate/pyruvate ratio and that it enhanced both the basal- and the glucagon-inhibited glucose synthesis from pyruvate alone or from a low lactate/pyruvate ratio (23). The authors suggested that the differing effects of EGF on gluconeogenesis depending on the substrate and the cytosolic and mitochondrial redox state as reflected by the lactate/pyruvate and 3-hydroxybutyrate/acetoacetate ratio (23, 24). Additionally, Neuregulin1α (Nrg1α) has been shown to suppress hepatic gluconeogenesis via the ERBB3-AKT/ERK-FoxO1 cascade (25). These divergent findings from isolated rat hepatocytes indicate that the hepatic impact of EGF is highly sensitive to the cellular metabolic environment; however, the lack of definitive *in vivo* data leaves its net contribution to systemic glucose production currently unresolved.

## Other EGFR ligands with distinct properties in glucose homeostasis

Although various ligands can activate EGFR, the distinctive phenotypes observed in animal models lacking specific EGFR ligands indicate that each ligand engages EGFR in a unique manner. This engagement contributes to the cell- and tissue-specific actions of both the receptor and its ligands, including their role in glucose homeostasis. In the following sections, we provide a summary and discussion of the properties exhibited by other EGFR ligands in relation to glucose homeostasis, organized chronologically by their year of discovery.

### Transforming growth factor α

Transforming growth factor α (TGFα) was initially discovered in the conditioned media of sarcoma cells and was originally named ‘sarcoma growth factor’ in 1981. It was later renamed ‘transforming growth factor (TGF)’ when similar activities were identified in other transformed cells and tumors (26). The ‘α’ designation was introduced to distinguish it from TGFβ, a completely unrelated protein (27).

TGFα serves as an important growth factor for islets, particularly during embryonic development and injury-induced regeneration (28). Overexpression of TGFα has been shown to induce the expansion of a PDX1 (pancreatic and duodenal homeobox 1)-expressing epithelium, characterized by focal expression of PAX6 (Paired Box 6) and initiation of islet neogenesis (29). These findings suggest that the premalignant events triggered by TGFα in the mouse pancreas may resemble a developmental program that is active during embryogenesis (29). In the liver, however, TGFα appears to act as a counter-regulatory factor. Evidence in rat hepatocytes suggests TGFα inhibits glycogen synthesis and counteracts insulin-induced glycogen deposition (30), potentially explaining the transient glucose intolerance observed during states of high liver regeneration or sepsis. As TGFα shows regenerative promise in the pancreas but potential inhibitory effects on hepatic glucose storage, the careful tissue-specific consideration in metabolic studies are required.

Interestingly, high glucose conditions upregulate TGFα transcription in rat aortic smooth muscle cells, indicating that TGFα is a glucose-responsive gene (31). This effect appears to be mediated by the hexosamine biosynthesis pathway rather than by glucose directly, with glutamine: fructose-6-phosphate amidotransferase (GFAT) serving as the key rate-limiting step (31, 32). In support of this mechanism, inhibition of GFAT markedly attenuates the glucose-induced increase in TGFα expression, while glucosamine, a downstream product of this pathway, induces TGFα even more potently than glucose. In this context, glucose-driven upregulation of TGFα may help amplify EGFR signaling in response to altered metabolic conditions, thereby contributing to changes in cell behavior relevant to glucose homeostasis.

### Amphiregulin

Amphiregulin (AREG) was initially discovered in the conditioned medium of a human breast carcinoma cell line in 1988 (33). Amphiregulin is synthesized as a precursor protein that remains anchored to the cell membrane, allowing it to engage in juxtacrine signaling with neighboring cells (34). Following proteolytic processing, amphiregulin is cleaved and released, functioning as either an autocrine or paracrine factor (35). Studies on transgenic mice expressing amphiregulin specifically in white adipose tissue have demonstrated reduced adipose tissue mass (36).

iRhoms, a group of proteins closely associated with rhomboid intramembrane proteases, lack catalytic activity. Instead, they are involved in regulating the activity of proteinase TACE, which plays a role in cleaving membrane-bound inflammatory cytokines like amphiregulin during their trafficking process (37). In mice lacking iRhom2, there is an accelerated gain of fat accompanied by insulin resistance when fed a high-fat diet (38). This phenotype is not associated with low-grade inflammation, as macrophage infiltration and tumor necrosis factor (TNF) production are decreased in the adipose tissue of HFD iRhom2-deficient mice (38). Notably, the induction of amphiregulin is completely absent in these iRhom2-deficient mice, suggesting a potential dominant role of amphiregulin in modulating insulin sensitivity via an EGFR-dependent mechanism (38). Taken together, these findings indicate that amphiregulin has emerged as a mediator of metabolic adaptation in adipose tissue.

Notably, AREG also facilitates post-RYGB glycemic control by upregulating GLUT1 and promoting intestinal glucose excretion (39). Mechanistically, RNA sequencing in RYGB-operated rats confirms that these effects are mediated through the activation of the EGFR/mTOR/AKT/GLUT1 signal transduction pathway (39). To further explore the role of amphiregulin in this context, obese or *ob/ob* mice were treated with amphiregulin (39). Notably, both systemic and local administration of amphiregulin led to an upregulation of GLUT1 transporters on the apical and basolateral membranes of small intestinal enterocytes (39). This increase in GLUT1 expression facilitated the excretion of glucose into the gut lumen, effectively preventing the exacerbation of hyperglycemia and contributing to the maintenance of serum glucose levels (39).

Interestingly, regulatory T cells (Tregs) also secrete amphiregulin following tissue injury to mediate repair and suppress inflammation (40, 41). This function led to the suggestion that AREG might protect against autoimmune diabetes by facilitating beta-cell repair. Contrary to this hypothesis, AREG deficiency in NOD mice failed to accelerate the disease, suggesting that endogenous AREG plays a limited role in preventing autoimmune progression (42). Consequently, the potential efficacy of pharmacological AREG supplementation warrants further investigation.

### Heparin-binding EGF-like growth factor

Heparin-binding EGF-like growth factor (HB-EGF) was first identified in 1991 as a heparin-binding protein secreted by macrophage-like cells (43). It was recognized as a member of the EGF family due to its structural similarity to EGF (44). HB-EGF is a heavily glycosylated EGF family member showing heparin affinity (45). The name “heparin-binding EGF-like growth factor” reflects its unique characteristic of binding to heparin, which distinguishes it from other EGF family members. Similar to other members of the EGF ligand family, HB-EGF is initially synthesized as a transmembrane protein called proHB-EGF (46). This proHB-EGF can be cleaved by metalloproteinases, resulting in the release of a soluble and diffusible ligand known as sHB-EGF (46).

Studies using carefully designed transgenic mouse models have shown that the ratio of cleaved to intact HB-EGF is more critical than the total amount of HB-EGF expressed in beta cells (46). The supporting evidence includes: First, although proHB-EGF is efficiently cleaved in islets, the overexpression of full-length proHB-EGF in beta cells resulted in 90% of the mice maintaining normal blood glucose levels (46). Second, it has been demonstrated that overexpressing soluble HB-EGF (sHB-EGF) in the beta cells of mice reduces blood glucose levels more rapidly compared to non-transgenic control mice (46). Third, an increase in uncleavable proHB-EGF in beta cells impairs the ability of mice to clear glucose from the blood following a glucose challenge by decreasing GLUT2 levels (46).

An intriguing study has demonstrated that a combination of soluble HB-EGF formulated with protected graft copolymers (PGC) and omeprazole—a proton pump inhibitor that reduces gastric acid secretion—can normalize fasting blood glucose, enhance islet function, and reduce insulitis in multiple low-dose streptozotocin-induced diabetic mice (47). Furthermore, the evaluation of various tissues (stomach, pancreas, kidney, liver, and spleen) indicated that the 28-day treatment did not cause neoplasms (47). The authors suggested that the soluble form of HB-EGF used in this study lacks an intracellular domain, which can initiate a self-sustaining signal for tumor formation (47). These findings underscore the promising translational potential of modified sHB-EGF for diabetes management while mitigating the risk of tumorigenesis.

In addition to HB-EGF mRNA levels are increased in β-cells in response to glucose in a carbohydrate response element-binding protein (ChREBP)-dependent manner, and this pathway is required for glucose-induced pancreatic β-cell proliferation in rats (48). HB-EGF has also been reported to be upregulated in skeletal muscle in response to exercise in mice (49). Transgenic mice overexpressing proHB-EGF in muscle exhibited a higher respiratory quotient compared to wild-type mice during indirect calorimetry, indicating their preference for carbohydrate as an energy substrate rather than fat (49). These animals also demonstrated substantial improvements in glucose tolerance, insulin sensitivity, and glucose uptake by skeletal muscle (49). They proposed HB-EGF produced by contracting muscle acts as an insulin sensitizer, facilitating the disposal of glucose in peripheral tissues (49). Furthermore, mice with high levels of transgene expression were largely protected from obesity, hepatic steatosis, and insulin resistance, even when fed a high-fat diet (49). In summary, HB-EGF is currently one of the most promising ligands for improving peripheral insulin sensitivity, though human validation is pending.

### Betacellulin

Betacellulin (BTC) was initially purified from the conditioned medium of a murine pancreatic beta cell carcinoma cell line in 1993 (50). Betacellulin is predominantly expressed in pancreas (51, 52), has shown potential in various pre-clinical studies related to type 1 diabetes treatment. *In vitro* experiments demonstrated that betacellulin can convert acinar cells into insulin-producing cells (53). Transplantation with betacellulin-transduced islets extends islet survival and preserves functional islet mass (54). However, it appears that betacellulin is not crucial for the regulation of insulin production by isolated pancreatic beta cells (52).

In animal studies, daily administration of recombinant human betacellulin via subcutaneous injections improved glucose tolerance in alloxan-induced type 1 diabetic mice (51). This effect was attributed to an increase in beta-cell volume primarily through accelerated neogenesis from ductal lining cells. Furthermore, betacellulin administration for approximately two weeks has shown the ability to improve glucose metabolism by promoting beta cell regeneration in 90% pancreatectomized rats (55) and mice with severe diabetes induced by a high dose of STZ (56). The underlying mechanism of betacellulin’s action involves inducing the expression of two key transcription factors Pdx-1 and islet factor 1 (Isl-1), which drive the differentiation of cells into insulin-producing cells (57). Various approaches have been explored in the context of type 1 diabetes, including the expansion and redifferentiation of pancreatic islet cells (54, 58–69). For instance, administration of a recombinant adenovirus expressing betacellulin into obese diabetic *db/db* mice has shown improvements in hyperglycemia (70).

Despite these promising findings on gene therapy with betacelluin and neurogenin 3 can reverse major metabolic problems in insulin-deficient diabetic mice (66), it is important to note that these findings are still limited to preclinical studies, and no clinical trials involving betacellulin have been conducted to date. This may be because betacellulin is considered a potent mitogen, its translational progress is hampered by its broad mitogenicity, which poses a significant risk for systemic tumorigenesis, stalling its transition to clinical trials.

### Epiregulin

Epiregulin (EREG), an insulin-sensitive factor, was identified through microarray technology using RNA samples from normal 3T3-L1 adipocytes and insulin-resistant 3T3-L1 adipocytes treated with tumor necrosis factor-alpha in 1998 (71). Subsequent studies have shown that, in the presence of glucose or arginine, treatment with epiregulin can influence the proliferation and secretory function of rat insulinoma INS-1E and RINm5F cell lines through the activation of the EGFR/ErbB1 pathway (72). Thus, epiregulin appears as a growth and insulinotropic factor in pancreatic beta cells.

Furthermore, a recent study uncovered a unique metabolic profile through crosstalk between epiregulin and the leptin receptor (LepR) (73). Epiregulin was shown to regulate glucose metabolism under leptin-deficient conditions in Lep^ob^ mice, but this effect was strictly dependent on the presence of LepR. Mechanistically, epiregulin stimulation of glucose uptake via the activation of the ERK/PI3K signaling pathway (73). Interestingly, it has been reported that epiregulin stimulates leptin secretion in HFD-fed mice (74). Treatment with recombinant epiregulin significantly enhances leptin production and secretion in a dose-dependent manner in intra-abdominal (iAb) white adipose tissue explants, primarily through activation of the EGFR/MAPK signaling pathway (74). Furthermore, the exogenous epiregulin upregulates genes involved in oxidative phosphorylation and thermogenesis at iAb fat pad, with these effects being strictly dependent on leptin signaling (74). Collectively, these findings highlight a critical crosstalk between epiregulin and leptin, wherein epiregulin compensates for leptin deficiency by enhancing leptin secretion. This positions epiregulin as a promising therapeutic target for addressing metabolic disorders associated with hypo- or dysregulated leptin signaling.

### Epigen

Epigen (EPGN), also referred to as epithelial mitogen, is the most recently identified ligand of the epidermal growth factor (EGF) receptor. It was discovered in 2001 through high-throughput sequencing of a mouse keratinocyte complementary DNA library due to its homology with other members of the EGF family (75). Two proteolytic cleavage events were found that release the segment harboring the EGF-like domain (76). It was proposed that the membrane-flanking site of the epigen active form is cleaved by membrane-anchored metalloprotease ADAM17 (77). Although epigen is claimed to be the last identified EGFR ligand, it was already discovered over 20 years ago. Compared to other EGFR ligands, its research and publication record remains notably limited. Many of these publications merely mention epigen as one of the genes included in other omics studies (75, 77–86). Very few studies have investigated its function and biological role in depth.

The phenotype of *Epgn* global knockout (KO) mice was firstly reported in 2013 (84), revealing that the mice are viable and do not exhibit any obvious phenotype. This means that the genetic deletion of *Epgn* does not affect mouse development, fertility, or organ physiology (84). The authors proposed that it might be due to functional compensation by other EGFR ligands (84). It was proposed that epigen is mitogenic for fibroblasts and epithelial cells based on its low-affinity and broad-specificity to ErbB family (76). Recombinant epigen could stimulate the proliferation of human epidermal keratinocyte HaCaT cells *via* c-erb-B1 receptors (75), while knockdown epigen could reduce metastatic outgrowth in mouse and human breast cancer models (78). However, the first transgenic mice overexpressing *Epgn* demonstrated peripheral demyelination in 2013 (85), and sebaceous gland hyperplasia in 2014 (86), meanwhile no cancer-related phenotypes were reported. Interestingly, it was reported that recombinant epigen suppresses extrusion to provide protective effects against fungal invasion using zebrafish (81).

A recent study by Chan et al. (2025) reported that *Epgn* knockout mice exhibit worsened glucose tolerance under HFD, while *Epgn* overexpression via adenovirus improves insulin secretion and suppresses hepatic gluconeogenesis (87). Short-term recombinant epigen injections improved glucose tolerance in both HFD-fed and STZ-treated mice via insulin-dependent (increased insulin secretion) and insulin-independent pathways (increased adipocyte and muscle glucose uptake and reduced hepatic gluconeogenesis) (87). Long-term low-dose epigen injections reduced fat mass, increased lean mass, enhanced adipocyte glycolysis and respiration, alleviated hepatic steatosis, and improved glucose and pyruvate tolerance in DIO mice (87). Long-term epigen treatment also promoted pancreatic β-cell regeneration, increased β-cell mass, and elevated insulin levels in STZ-treated mice (87). However, it must be emphasized that these findings represent emerging evidence from specific rodent models. Independent confirmation by other research groups and a more detailed investigation into the long-term safety profile of EPGN are required before its therapeutic relevance to human diabetes can be established.

## ErbB4 and EGFR ligands mediated glucose metabolism

Interestingly, few EGFR ligands, namely HB-EGF, epiregulin, and betacellulin, are also active on ErbB4 (also named as HER4) receptor (**Table 1**). Genetic studies have suggested a link between ErbB4 and type 2 diabetes as well as obesity (88). ErbB4 is mainly expressed in skeletal muscle and adipose tissues (88). Mice with ErbB4 deletion developed metabolic syndrome after 24 weeks on a medium-fat diet (MFD), as evidenced by obesity, dyslipidemias, hepatic steatosis, hyperglycemia, hyperinsulinemia, and insulin resistance, compared to wild-type mice (88). Additionally, ErbB4 deletion mice exhibited increased subcutaneous and visceral fat with severe inflammation. ErbB4 deficiency-related obesity and adipose tissue inflammation may play a role in the development of the metabolic syndrome (88). Furthermore, ErbB4 expression in pancreatic beta cells supports their survival and enhances insulin secretion, protecting against dysfunction that can lead to diabetes (89).

**Table 1:** Metabolic impact and evidence level of EGFR ligands.

Ligand	Target receptor	Primary metabolic effect	Primary model(s)	Evidence level	Signaling context
EGF	EGFR	↑ Insulin secretion; β-cell survival	*In vitro* (MIN6), Rodent	High	Soluble ligand; Acute response
TGF**α**	EGFR	β-cell neogenesis; ↓Hepatic glycogen	Rodent	Moderate	Membrane-bound precursor
HB-EGF	EGFR, ErbB4	↑ Muscle glucose uptake; ↑↑Insulin sensitivity	Rodent	High (Preclinical)	sHB-EGF vs proHB-EGF ratio
BTC	EGFR, ErbB4	β-cell neogenesis/regeneration	Rodent	High (Preclinical)	Pdx-1/Isl-1 activation
EREG	EGFR, ErbB4, LepR	↑Glucose uptake; ↑ Leptin secretion	Rodent (*ob/ob*)	Emerging	Receptor cross-talk (LepR)
AREG	EGFR	Adipose insulin sensitivity; Intestinal glucose excretion	Rodent (RYGB)	Moderate	iRhom2-dependent shedding
EPGN	EGFR	↓Gluconeogenesis; ↑ β-cell mass	Rodent (HFD/STZ)	Emerging	Low-affinity, sustained signaling

## ErbB4 ligands and EGFR-medicated glucose metabolism

In addition to the few EGFR ligands discussed above, certain ligands can activate ErbB4 receptor. Neuregulins (NRGs) constitute a family of signaling proteins that primarily engage ErbB4, thereby regulating a range of biological processes, including glucose metabolism (90). The neuregulin family includes several members, such as NRG1, NRG2, NRG3, and NRG4 (91). Like EGFR ligands, neuregulins contain an EGF-like domain, which is essential for receptor binding (91). However, unlike EGFR ligands, neuregulins do not bind directly to EGFR (91). Instead, they can indirectly activate EGFR by forming heterodimers with other ErbB family members, and activation of downstream signaling pathways such as PI3K-Akt and MAPK, which are critical for metabolic regulation (91). For instance, NRG4, an important ErbB4 ligand highly expressed in adipose tissue, has been shown to improve insulin sensitivity, suppress adipose inflammation, regulate lipid metabolism, and modulating brown adipose tissue activity, thereby indirectly stabilizing glucose homeostasis (92). In the liver, NRG4-ErbB4 signaling reduces hepatic lipogenesis, further contributing to improved glucose regulation (92). Although one study reported that NRG1 treatment in diabetic mice increased EGFR phosphorylation (93), the primary metabolic improvements were specifically linked to ErbB3/ErbB4 pathways (94).

## Controversial effects of EGFR tyrosine kinase inhibitors in glucose regulation

In contrast to EGFR ligands, EGFR tyrosine kinase inhibitors (TKIs) exhibit controversial effects on glucose regulation, with evidence supporting both hyperglycemia induction and improvements in glucose tolerance. Given the critical roles of downstream signaling pathways, particularly AKT and mTOR, in maintaining glucose homeostasis, it was anticipated that EGFR inhibitors would similarly lead to hyperglycemia, as observed with other TKIs (95, 96). However, this adverse effect is not universal across all EGFR TKIs; in fact, most EGFR TKIs do not significantly impact glucose homeostasis (96).

For example, preclinical studies involving the EGFR inhibitor PD153035 have demonstrated enhancements in glucose tolerance and insulin sensitivity in mouse models fed high-fat diets (92). These improvements are linked to reductions in inflammation, macrophage infiltration in adipose tissue, and enhanced insulin signaling pathways (92). This suggests that improved glucose homeostasis may be indirectly mediated by lower levels of pro-inflammatory cytokines and improved insulin receptor function, rather than through direct regulation of glucose uptake.

However, third-generation agents, such as rociletinib, have been clinically associated with hyperglycemia in patients with non-small cell lung cancer (NSCLC) (6). Notably, rociletinib appears to be particularly implicated, largely due to its metabolites M460 and M502, which are potent inhibitors of the insulin receptor (IR) and insulin-like growth factor-1 receptor (IGF-1R) (97). These metabolites are thought to promote hyperglycemia by disrupting IR/IGF-1R–mediated signaling pathways essential for glucose metabolism, rather than through the pharmacologic activity of the parent compound. Furthermore, humans generate substantially higher circulating levels of M460 and M502 than mice or rats (96). This species difference in metabolite exposure may help account for the discrepancies observed between preclinical animal findings and human clinical outcomes.

In summary, these observations highlight that the varying chemical properties and specificities of EGFR inhibitors and characteristics of different subjects can significantly influence glucose regulation outcomes, thereby emphasizing the ongoing controversy surrounding EGFR inhibitors.

## Limitations of using EGFR ligands for glucose homeostasis

In summary, these findings suggest that EGFR ligands could represent a potential therapeutic target for managing glucose homeostasis and metabolic disorders, including type 2 and type 1 diabetes. However, there are several challenges to consider when exploring the use of EGFR ligands for glucose regulation.

### Off-target effects

EGFR is expressed in wide range of tissues and EGFR signaling is not limited to glucose metabolism but is involved in various cellular processes. Modulating EGFR signaling may have systemic effects on other organs and biological functions. It is crucial to assess potential impacts on overall health and organ systems when considering the use of EGFR ligands for glucose homeostasis. However, many EGFR ligand activation of downstream signaling pathways via interactions with other receptors such as ErbB4 for HB-EGF and betacellulin, and Leptin receptor for epiregulin. *In vivo* experiments need to be performed to exam the severity of the potential off-target effects.

### Safety concerns

Constitutive or ligand-independent signaling of EGFR has largely been considered a property of oncogenicity (98). The effects of EGFR ligands on glucose homeostasis may be temporary or short-lived. Continuous administration or repeated dosing may be required to sustain the desired metabolic effects. However, long-term safety and potential adverse effects of prolonged EGFR ligand use need to be carefully monitored. For instance, EGFR signaling plays a critical role in various cellular processes, including cell proliferation and survival. Modulating EGFR activity with ligands carries the risk of promoting abnormal cell growth, potentially leading to tumorigenesis. Therefore, a comprehensive assessment of the safety profile, supported by rigorous preclinical and clinical studies, is essential to mitigate the risk of adverse effects.

For example, fibroblast growth factors, particularly FGF21 and FGF19, have emerged as promising candidates in diabetes treatment through their roles in metabolic regulation (99). While FGF19 shows promise in metabolic regulation and diabetes treatment, its role in cancer progression is a critical concern. FGF19, when overexpressed, can activate the FGFR4 receptor, triggering pathways involved in cell proliferation, survival, and migration, which are linked to tumor growth in various cancers, such as hepatocellular carcinoma (100). To address this, FGF19-based analogues are being engineered to retain their metabolic benefits while avoiding activation of tumorigenic pathways (101). This approach aims to balance the therapeutic potential of FGF19 with the need to minimize its oncogenic risks. A similar approach can be applied to EGFR ligands by engineering analogues that selectively activate metabolic pathways without triggering oncogenic signaling.

## Future directions: chimeric ligands and targeted delivery

To circumvent the oncogenic risks associated with constitutive EGFR activation, future research should explore biased signaling or chimeric ligands. For example, engineering dual-specific EGFR/ErbB4 ligands could potentially harness the insulin-sensitizing effects of the ErbB4 axis (similar to Neuregulin-4) while utilizing the regenerative capacity of the EGFR axis. This dual-agonist framework builds upon the highly successful clinical paradigm of GLP-1/GIP chimeric peptides—exemplified by the breakthrough multi-receptor agonist tirzepatide—which has redefined the therapeutic landscape of metabolic disease (102, 103). The next-generation of EGFR biomimetics must be engineered for signaling bias, preferentially activating metabolic cascades (PI3K/AKT) over mitogenic pathways (MAPK/ERK) to achieve a superior safety and efficacy profile.

## Conclusion

**Figure 1:**
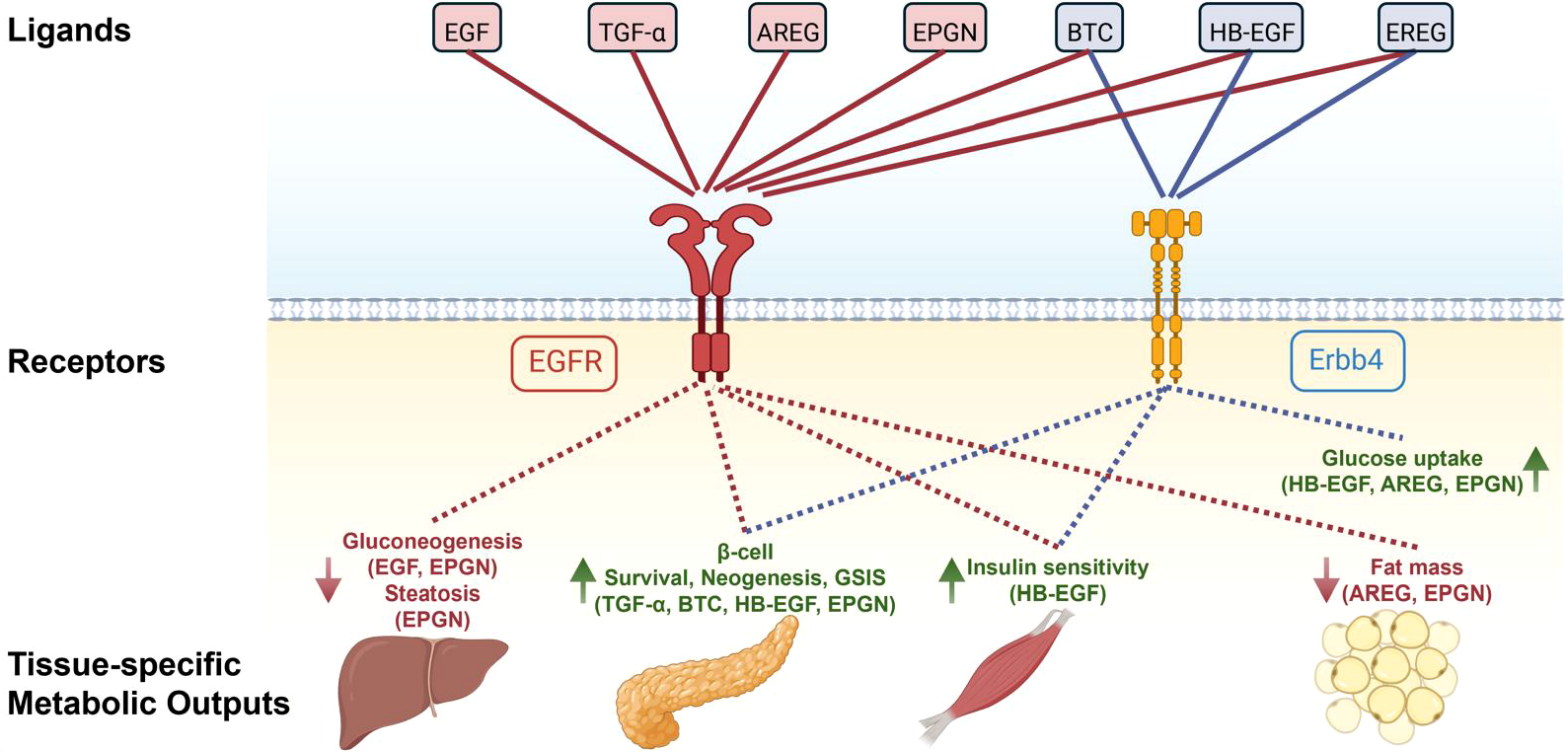
Diagram illustrating signaling pathways of EGFR and Erbb4 receptors activated by various ligands, showing liver, pancreas, muscle, and fat-specific metabolic outputs including gluconeogenesis, steatosis, beta-cell survival, insulin sensitivity, glucose uptake, and fat mass changes.

The EGFR signaling axis represents a potent, yet complex, regulator of glucose homeostasis (**Figure 1**). Preclinical data provide a robust mechanistic foundation for the roles of all seven ligands, particularly in beta cell regeneration and peripheral insulin action. However, the field currently lacks definitive human evidence, and the mitogenic risk of EGFR activation remains a formidable barrier. Reflecting on the advancements in the commonly using hormone-based diabetic therapies, such as insulin and GLP-1 analogues, numerous studies have raised concerns about their potential mitogenic effects, which could pose safety risks time to time in the past (104, 105). Future studies must move beyond descriptive rodent models toward the development of safer, biased-signaling analogues and tissue-specific delivery systems.
